# Antibacterial and Bioactive Surface Modifications of Titanium Implants by PCL/TiO_2_ Nanocomposite Coatings

**DOI:** 10.3390/nano8100860

**Published:** 2018-10-20

**Authors:** A. Sandeep Kranthi Kiran, T.S. Sampath Kumar, Rutvi Sanghavi, Mukesh Doble, Seeram Ramakrishna

**Affiliations:** 1 Medical Materials Laboratory, Department of Metallurgical and Materials Engineering, Indian Institute of Technology Madras, Chennai 600036, India; urskranthi.kiran@gmail.com; 2 Department of Biotechnology, Bhupat and Jyoti Mehta School of Biosciences, Indian Institute of Technology Madras, Chennai 600036, India; rutvirs93@gmail.com (R.S.); mukeshd@iitm.ac.in (M.D.); 3 NUS Centre for Nanofibers and Nanotechnology, Department of Mechanical Engineering, National University of Singapore, 2 Engineering Drive 3, Singapore 117581, Singapore

**Keywords:** titanium, antibacterial coatings, electrospinning, nanocomposite coatings, TiO_2_ photocatalytic, orthopedic infections

## Abstract

Surface modification of biomedical implants is an established strategy to improve tissue regeneration, osseointegration and also to minimize the bacterial accumulation. In the present study, electrospun poly(ε-caprolactone)/titania (PCL/TiO_2_) nanocomposite coatings were developed on commercially pure titanium (cpTi) substrates for an improved biological and antibacterial properties for bone tissue engineering. TiO_2_ nanoparticles in various amounts (2, 5, and 7 wt %) were incorporated into a biodegradable PCL matrix to form a homogeneous solution. Further, PCL/TiO_2_ coatings on cpTi were obtained by electrospinning of PCL/TiO_2_ solution onto the substrate. The resulted coatings were structurally characterized and inspected by employing scanning electron microscope (SEM), X-ray diffraction (XRD), and Fourier transform infrared (FTIR) spectroscopy. Given the potential biological applications of PCL/TiO_2_ coated cpTi substrates, the apatite-forming capacity was examined by immersing in simulated body fluid (SBF) for upto 21 days. Biocompatibility has been evaluated through adhesion/proliferation of hFOB osteoblast cell lines and cytotoxicity by MTT assay. Antimicrobial activity of PCL/TiO_2_ nanocomposites has been tested using UV light against gram-positive Staphylococcus aureus (*S.aureus*). The resulting surface displays good bioactive properties against osteoblast cell lines with increased viability of 40% at day 3 and superior antibacterial property against *S.aureus* with a significant reduction of bacteria to almost 76%. Surface modification by PCL/TiO_2_ nanocomposites makes a viable approach for improving dual properties, i.e., biological and antibacterial properties on titanium implants which might be used to prevent implant-associated infections and promoting cell attachment of orthopedic devices at the same time.

## 1. Introduction

Biocompatible titanium (Ti) and its alloys are broadly accepted metallic materials for hard tissue repair (orthopedic and dental) for its exceptional combination of biomedical and mechanical properties [1]. Even though Ti and its alloys are used as an implant material for more than three decades, there are still some inadequacies that need to be addressed. Especially, bacterial associated diseases/infections during surgery always carry serious hazards leading to a severe clinical economic consequence such as re-hospitalization, complex re-surgeries, implant loosening, high economic associated costs and sometimes even death. Recent studies estimated the current incidence of bacterial infection had incurred a total financial cost of $10 billion with close to 100,000 infections and 8000 reported deaths in the United States alone [2,3]. The reason being, when compared to bioactivity enhancement modifications, relatively very few efforts have been made to address antibacterial activity on the surface before the implantation. It is a known fact that treating an infected orthopedic implant materials post-surgery is hugely complicated, primarily due to the inherent difficulties of treating an established biofilm formed by microorganisms on the surface [4].

Nevertheless, many preventative strategies have been proposed by academics to improve the antibacterial ability of the material before the implantation/surgery [5,6]. But, most of the solutions proposed for obtaining antibacterial surfaces without losing its bioactivity require a complex coatings technique. In this view, several novel strategies such as topographical modifications (nanotubes), incorporating antibacterial agents (Ag, Cu) and various surface treatments [7,8,9,10] were suggested/developed to disinfect the bacterial colonization on biomedical implants before the implantation. However, still traces of evidence of bacterial invasion can be still found even after the post-surgery. Nevertheless, these findings highlight the crucial need modifications to the material to prevent bacterial implant-associated infections at early stages. Among these, surface modification either by treatment or coating on the implant material has been well recognized as the best substitute to design and alter the biological performance of the Ti and its alloys [11].

Titanium dioxide (titania, TiO_2_), a bioceramic material have become a focus of significant research due to its versatile characteristics [12,13]. TiO_2_ nanoparticles are well-known for its stability, non-toxicity, UV resistance and found its application in cosmetics, electronics, biomedical, optics and also as a cleaning reagent [14]. Ever since first reported by Matsunaga et al., TiO_2_ photocatalytic properties have drawn more attention in the biomedical field for its specific ability to a kill wide variety of microorganisms under a strong UV radiation [15,16]. In brief, when the surface of TiO_2_ is exposed to a strong UV light, electron-hole pairs (*e*^−^-*h*^+^) are generated in the valence band and reacts with oxygen and atmospheric water (OH^−^) thereby yielding to reactive oxygen species (ROS). The generated ROS acts a powerful oxidizing agent capable of decomposing organic molecules and inactivating micro-organisms through a series of chemical reactions, leading to the powerful antibacterial agent [17,18,19]. Also, for enhancing the composite cell attachment and proliferation properties, TiO_2_ nanoparticles are projected as a secondary phase material for biodegradable polymer matrices [20,21,22,23,24,25].

Over the past few years, polymer/ceramic nanocomposites as scaffold materials have attracted more attention for bone tissue engineering. Many works have been described in the literature which explains the enhancement of Ti-based implants with organic or organic-inorganic substitute surface coatings [26,27,28]. In particular, because of the high porosity and large specific surface area, nanocomposite fiber scaffolds have been successfully explored in tissue engineering for orthopedic implants. Among the various techniques for nanocomposite scaffold fabrication, the electrospinning process is described as the most reliable process for producing long and continuous fibers. Electrospinning is a simple and economical fiber fabrication technique that utilizes electrical forces to produce ultrafine micro and nanofibers templets with a wide range of polymers for a variety of applications [29,30]. In most typical tissue engineering strategies, the engineered 3D porous scaffolds serve as a pattern for cell adhesion, expansion, and proliferation of cells ingrowth. Electrospun nanofibers are favorably proficient of imitating microarchitecture of native ECMs owing to their high surface area to volume ratio and relatively large internal porosity. This technique also enables to entrap inorganic ceramic nanoparticles into the organic polymer in a very convenient way to enhance physical, chemical and mechanical properties.

Polycaprolactone (PCL), a semi-crystalline biodegradable polymer, known for its superior mechanical properties, excellent biocompatibility, and slower degradation rate. It is a widely accepted polymer for drug release carriers, biodegradable packaging materials, and more importantly for the development of 3D scaffolds for bone tissue engineering applications. Numerous techniques have been developed to fabricate PCL-based scaffold to a simple two-dimensional structure (casting) to complex three-dimensional (3D printing) objects [31,32]. However, PCL in current form is hydrophobic, which results in lack of wettability and poor cell attachment. Successful blending with bioceramics haven been reported elsewhere [9,23,27,33] for improved biological properties.

The current study aims to incorporate TiO_2_ nanoparticles into the PCL scaffolds for improving mechanical properties, biological properties (bioactivity, anti-bacterial, cell adhesion, and cell proliferation) and physiochemical properties (hydrophilicity) for orthopedic applications. To achieve that, PCL/TiO_2_-nanocomposite scaffolds were synthesized by electrospinning and coated on cpTi substrates.

## 2. Materials and Methods

### 2.1. Sample Preparation

Commercial pure titanium (cpTi), biomedical grade 2 (0.015% carbon, 0.1156% oxygen, 0.0095% nitrogen, 0.0013% hydrogen, 0.04% iron) plates with dimensions of 10 mm × 10 mm × 2 mm (MIDHANI, Hyderabad, India) were used as the base material. The substrates were polished using 300, 600, 800 and 1200 emery sheets followed by disc polishing to mirror finish using diamond paste. The polished samples were further degreased by cleanser, ultrasonically washed with ethanol and sonicated in acetone several times before drying in air overnight.

### 2.2. Synthesis of PCL/TiO_2_ Nanocomposites

PCL/TiO_2_ polymer-ceramic hybrid composites containing 2, 5 and 7 wt % of the ceramic content was synthesized by means of the electrospinning process. In brief, poly(ε-caprolactone) (PCL, 80 kDa; Sigma-Aldrich, Singapore) pellets were dissolved in chloroform and methanol (Sigma-Aldrich, Singapore) at 3:1 ratio to make an 8% (*w*/*v*) solution and kept under stirring conditions for 4 h. TiO_2_ was synthesized as reported earlier *via* electrospraying method by using titanium isopropoxide as the main precursor [34]. To prepare PCL/TiO_2_ nanocomposites with a certain PCL to TiO_2_ ratio, a known amount of TiO_2_ (2, 5 and 7 wt %) powders were added into the solution batch wise under string conditions. Because of the nature of TiO_2_ nanoparticles, it was very critical to homogeneously disperse TiO_2_ nanoparticles in the PCL matrix to acquire satisfactory dispersion without agglomeration. The mixed solution was under stirring conditions for 72 h using a magnetic stirrer with sonication for every 6 h. Upon attaining satisfactory homogeneous conditions, PCL/TiO_2_ solution was taken in a 5 mL syringe equipped with a 22-gauge rounded metal needle. A constant flow rate was set to optimized 500 µL/h through a syringe pump (NE1000, U.S.A). A constant high voltage of 12.0 kV was applied to the needle tip which was positioned at a distance of 10 cm from the grounded collector. Previously cut cpTi substrates of dimensions 10 mm × 10 mm × 2 mm were fixed on the aluminum foil as a collector. Prior to the coating procedure, the substrates were washed, sonicated in dry ethanol for 1 h and left overnight for drying. After the coating procedure, all the samples were dried under vacuum for 1 day to completely remove any solvent residues present.

### 2.3. Characterization

The morphology, phase analysis, and chemical composition of the obtained PCL/TiO_2_ coatings were characterized by scanning electron microscopy (SEM, Hitachi, S-4300, Tokyo, Japan) attached with energy dispersive X-ray spectroscopy (EDX, Thermo Noran, Sonora, CA, USA), X-ray diffractometer (XRD, D8 DISCOVER, Bruker, Billerica, MA, USA) and Fourier transform infrared spectroscope (FT-IR, Perkin-Elmer Spectrum Two spectroscopy) respectively. Morphological analysis of the surface before and after coatings were examined by SEM, operated at an accelerating voltage ranging from 10–15 kV. To avoid any ‘charging’ and increase conductivity, the dry substrates were sputter-coated for 30 s with a thin deposits of gold (Au) before observing in SEM. XRD (at 2θ = 10°–90°) is used for analyzing the phases present in the synthesized nanocomposites coatings with Cu-Kα radiation (λ = 1.540 Å) at a scanning rate of 0.1 step/s. To analyse the chemical composition, FT-IR measurements were performed over a range of 4000–500 cm^−1^. To identify functional groups of the synthesized samples, KBr powder is mixed and made of pellets. In addition, surface wettability was calculated by measuring static contact angles of deionized water with a contact angle system (Easy DROP, KRUSS, Stuttgart, Germany) at the ambient temperature. For each set of samples, a 10 µL drop of water was deposited on each substrate and allowed to rest for 5 s.

### 2.4. Biological Studies

#### 2.4.1. Biominerilization Studies

To evaluate the apatite-forming ability (bioactivity), the substrates were immersed in 30 mL of simulated body fluid (SBF) with material composition and composition nearly equal to human blood plasma for 14 days. The SBF solution is prepared in the laboratory by mixing laboratory-grade chemicals (NaCl, NaHCO_3_, KCl, K_2_HPO_4_·3H_2_O, MgCl_2_·6H_2_O, CaCl_2_, and Na_2_SO_4_) in deionized water and maintained at pH 7.4 with tris (hydroxy-methyl) aminomethane and 1 M HCl. The detailed information on ion concentrations of SBF proposed by Kokubo et al. without organic species and its correlation with human blood plasma can be found elsewhere [35]. The falcon tubes containing SBF and substrates were immersed in water bath, maintained at static conditions at 37 °C. After immersing for 14 days, the substrates were carefully taken out from the SBF solution, washed with deionized water and freeze dried at room temperature for further morphological and elemental analysis.

#### 2.4.2. Antibacterial Assay

Prior to inoculation, PCL/TiO_2_ samples with different TiO_2_ concentration were UV-irradiated for 5 h to stimulate the photocatalytic reaction in the TiO_2_ material. Other samples (pure PCL and cpTi substrate) were UV-irradiated and sterilized for the same time interval. These specimens were used for carrying out the antibacterial assay against bacterium Staphylococcus aureus (NCIM 5021). In brief, the samples were placed in 12 welled plates containing 2 mL nutrient broth. A bacterial inoculum of 1 × 107 CFU/mL was added to each well. The plates were then allowed to incubate for 24 h at 37 °C.

After 24 h the samples were carefully removed and rinsed. The samples were sonicated for one minute with an interval of one minute. Five cycles were repeated to ensure complete extraction of bacteria. These samples were centrifuged, and the bacterial pellet obtained, was resuspended in PBS. The bacterial solution was then spread onto pre-cooled agar plates and incubated for 24 h at 37 °C. The resulting colonies were then counted, and the log values were calculated for them. The reduction in bacterial growth was estimated as a reduction in log CFU/mL values

#### 2.4.3. Cell Culture Studies

The cytotoxicity, cell adhesion and proliferation of surface coated PCL/TiO_2_ nanocomposites were determined by using human fetal osteoblastic cell lines (hFOB 1.19, ATTC CRL 11372) for day 1 and day 3. Five substrates (cpTi, PCL with 2, 5 and 7 wt % of TiO_2_ content) were adopted to evaluate the potential of using them for biomedical applications. In brief, cells were cultured in base medium of hFOB cell lines i.e., Dulbecco’s Modified Eagle’s Medium/Ham’s Nutrient Mixture F12 (1:1 DMEM/F12) and complemented with 10% fetal bovine serum (FBS), 1% of non-essential amino acids (MEM) and antibiotics (penicillin G, and streptomycin). For the entire duration of experiment, the culture medium was replaced every alternative day and was preserved in a humidified incubator at 37 °C under an atmosphere of 5% CO_2_. After attaining about 80% confluence, the cells were trypsinated and digested at a final concentration of 5 × 10^4^ cells/cm^2^ onto the substrates in 24-well plates. Prior to cell seeding, the substrates with PCL/TiO_2_ coatings were sterilized by immersing in 70% ethanol for 1 h followed by washing 3 times with sterilized phosphate buffered saline solution (pH = 7.4, PBS).

MTT assay for toxicity connected with cell viability and proliferation were also observed on surface coated cpTi substrates for day 1 and day 3. Pure cpTi without any coating is used as the control. After removing the culture medium, hFOB cells were quantitatively assessed by seeding 4 mg mL^−1^ MTT 3-(4,5-dimethylazol-2-yl)-2,5-diphenyltetrazolium bromide (yellow) reagent on to the substrates and determined at day 1 and day 3. In brief, both days of incubation, culture medium was removed and washed with 400 µL of prewarmed PBS. Then 400 µL of culture medium accompanied with 60 µL of MTT solution was added to each well-plate containing samples and incubated at 37 °C in a 5% CO_2_ humidified atmosphere. After an incubation period of 4 h, 100 µL of the resulting supernatant was added to each well of 96-well ELISA plate. The plates were gently agitated for 3 min to establish complete crystal dissolution. Percentage cell viability was determined by recording optical absorbance at 570 nm with reference to 690 nm using a microplate reader (Bio TEK Instrument, Winooski, VT, USA, EL307C). To ensure reproducibility, tests were carried out by performing triplicates of samples.

#### 2.4.4. Cell Morphology

Samples for SEM analysis were withdrawn from culture after 3 days incubation. For cell adhesion and proliferation studies, before fixing with 3% glutaraldehyde solution, cell-seeded substrates were washed with PBS thrice to confiscate any separated cells. The samples were cleaned again with PBS after 30 min, then kept at 4 °C. After cell fixation, the substrates were dehydrated in a series of ethanol solutions at varying concentrations from 30% to 100% for 15 min each. 1 mL of hexamethyldisilane (HMDS) was added on each sample and left to dry for 2 days. Prior to SEM image analysis, all the samples were gold coated for 20 s.

### 2.5. Statistical Analysis

All data values are presented a mean ± standard deviation for each group of samples. A paired sample *t*-test and one-way analysis of variance (ANOVA) were performed to determine statistically significant differences between groups. All tests were conducted with 95% confidence intervals (*p*-value < 0.05).

## 3. Results and Discussion

### 3.1. Surface Characterization

**Figure 1:**
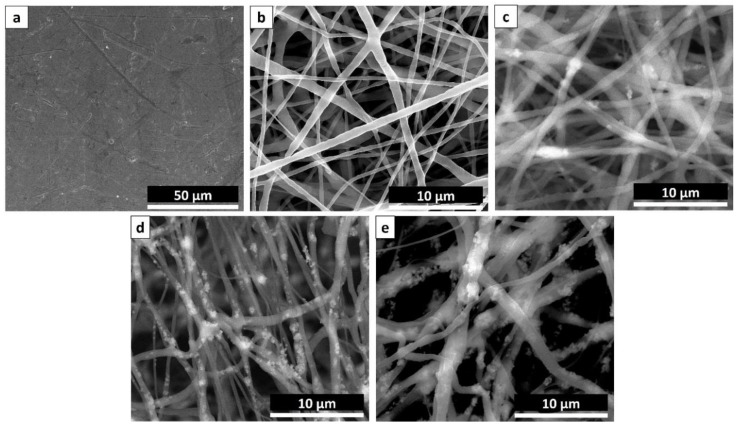
SEM Images displaying (**a**) cpTi substrate; PCL mat containing (**b**) 0 wt % TiO_2_, (**c**) 3 wt % TiO_2_, (**d**) 5 wt % TiO_2_, and (**e**) 7 wt % TiO_2_.

**Figure 2:**
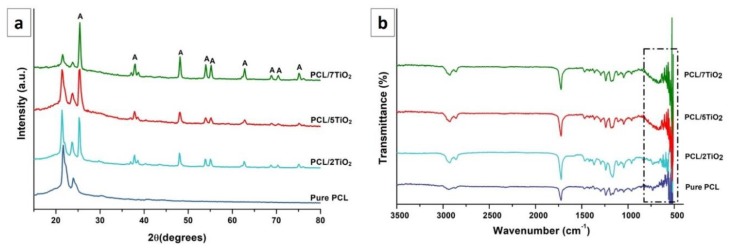
(**a**) XRD and (**b**) FTIR patterns of PCL/TiO_2_ (2, 5 and 7 wt %) nanocomposites in comparison with pure PCL.

**Figure 3:**
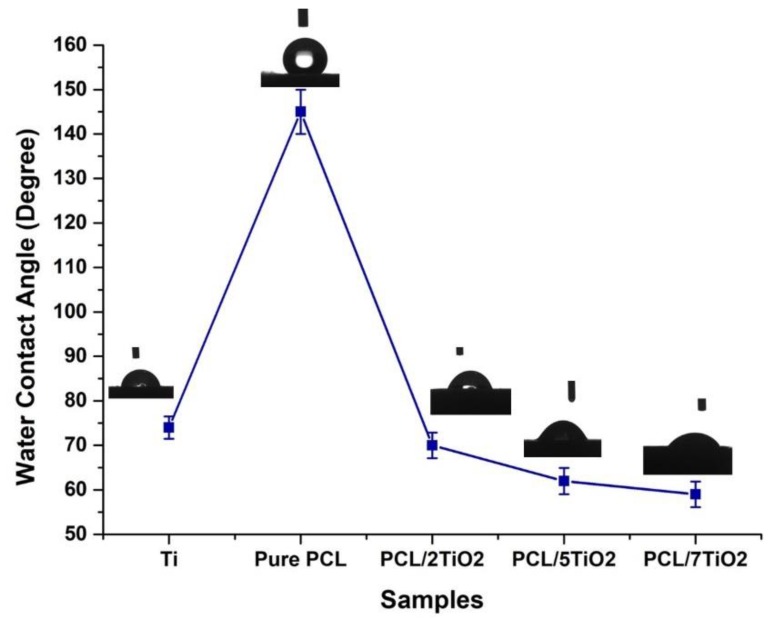
Water contact measurements on various surfaces obtained as a function of TiO_2_ wt % with distinctive water droplet images after 5 s from droplet.

**Figure 4:**
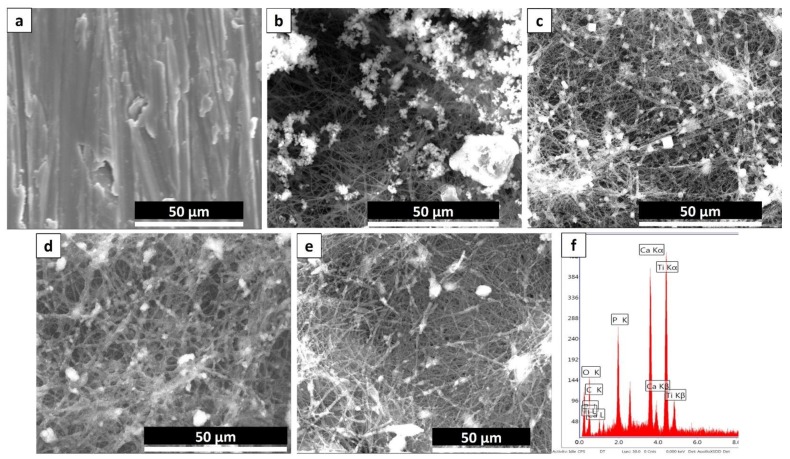
SEM images of (**a**) An, (**b**) PCL (**c**) PCL with 2 wt % TiO_2_ (**d**) PCL with 5 wt % TiO_2_ and (**e**) PCL with 7 wt % TiO_2_ after immersing in SBF for 21 days. (**f**) EDS analysis results for the newly formed calcium and phosphate of PCL with 5 wt % TiO_2_.

**Figure 5:**
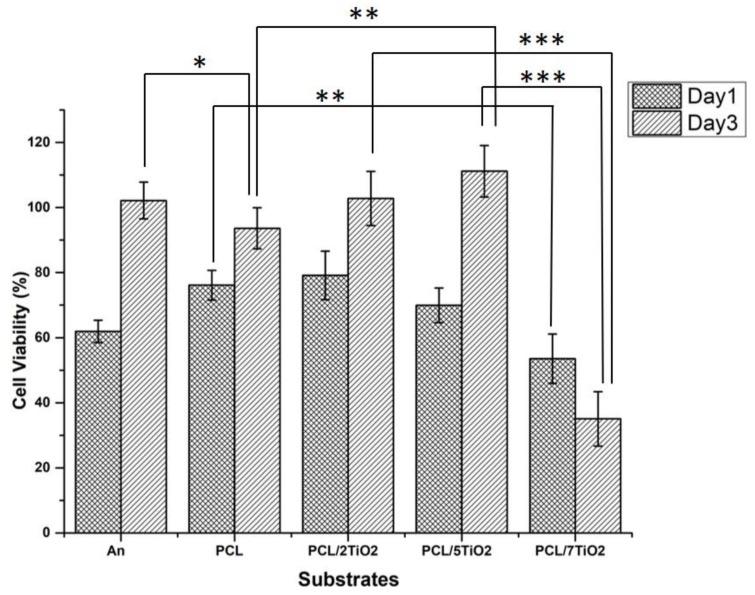
hFOB Cell viability on An, Pure PCL coated, PCL/2TiO_2_ coated, PCL/5TiO_2_ coated and PCL/7TiO_2_ coated cpTi samples cultured for day 1 and day 3 (*p* < 0.05).

SEM images of unmodified cpTi without any surface coating (Figure 1a) and PCL/TiO_2_ nanocomposites with and without TiO_2_ particulate additions were presented in Figure 1b–e respectively. Due to an exact balance between the solution viscosity and electrical conductivity, continuous uniformity of the fibrous structures without any beads were observed in all the samples. Figure 1b shows the pure PCL nanofibers (0 wt % TiO_2_) in uniform diameter with a smooth surface with an average diameter of 540 ± 40 nm. But, the morphology of the fibers was adversely affected by the addition of TiO_2_ nanoparticles. 8 wt % of PCL was used as the constant polymeric solution for electrospinning and a gradual growth in fiber diameter was observed when TiO_2_ content increased from 2 wt % (Figure 1c) to 5 wt % (Figure 1d) and to 7 wt % (Figure 1e). With the increase of TiO_2_ particle concentration, the size of nanofibers tends to become more significant and visible agglomeration to some extent were observed inside the fibers. For example, the average fiber diameter increases from 640 ± 60 nm to 710 ± 20 nm and 900 ± 89 nm for as-spun PCL/2TiO_2_, PCL/5TiO_2_ and PCL/7TiO_2_ composite nanofibers respectively. The results were in agreement with another study where the addition of nanoparticles increased the diameter of fiber [33].

Also, based on the SEM analysis of Figure 1c,d, the results also indicate that TiO_2_ nanoparticles are directly embedded inside the PCL nanofiber matrix rather than exposed on top of the fibers. The concentration of 7 wt % of ceramic TiO_2_ (Figure 1e) causes some of the nanoparticles surfaced on the as-spun nanofiber mats, indicating inhomogeneous mixing of the particles. In view of this, no further experiments above 7 wt % TiO_2_ have been conducted as it gives the notion that “*outer*” particles on fibers would be leached away easily as no chemical interaction with the PCL nanofibrous mat is made. Due to this, there may also be a negative effect on the mechanical properties (tensile strength and Young’s modulus) of the composite. Also, the morphologies of fibers became more irregular when the increasing of TiO_2_ content which it might because of the influence of ceramic particles on the solution viscosity, surface tension, and concentration. So, optimization of nanoparticle concentration in connection to the mechanical properties of the nanocomposite is sometimes essential.

### 3.2. Phase Analysis

The XRD patterns of PCL mat and PCL/TiO_2_ coatings with 2, 5 and 7 wt % of TiO_2_ content is shown in Figure 2a along with electrosprayed TiO_2_ nanoparticles for reference. PCL is a semi-crystalline polymer, which can be spotted by two characteristic peaks in the region of 20°–25° (2θ = 22.1° and 24.5°). The TiO_2_ anatase nanoparticles exhibit characteristic peaks at 2θ values of 25.6°, 35.9°, 37.9°, 38.9°, 48.4°, 53.9° and 56.1°, corresponding to the diffraction patterns of (101), (103), (004), (112), (200), (105), and (211) crystalline planes respectively (JCPDS data No. 36–1451). From the Figure 2a, it can be noted that the relative intensities of TiO_2_ are increased with increasing TiO_2_ content. Also, incorporation of TiO_2_ nanoparticles into the PCL fibrous structure resulted in the widening of peak widths and reduction in its peak height implying the decrease in the crystallinity of PCL structure.

FTIR spectra of the PCL/TiO_2_ coatings with 2, 5 and 7 wt % of TiO_2_ content and pure PCL mat for comparison are shown in Figure 2b. The analysis shows the presence of H-bonds between organic (PCL) and inorganic (TiO_2_) components of the hybrid materials. The spectrum of the TiO_2_ coatings are visible at wavenumber lower than 998 cm^−1^ are due to Ti-O and Ti-O-Ti vibration bands in the lattice [36]. The peaks located at 1725 cm^−1^, 1182 cm^−1^, 1054 cm^−1^, and 2865 cm^−1^ corresponds to carbonyl groups of C=O, C–O–C, C–O, and alkyl group of C=H stretching vibrations of PCL polymer respectively. The bands present at 564–647 cm^−1^ (PO_4_^3−^ bending vibration), 878 cm^−1^ (P–OH stretching) and 999–1102 cm^−1^ (PO_4_^3−^ asymmetric stretching) [37]. Most corresponding bands of TiO_2_ were detected in the PCL/TiO_2_ spectrum recommends that TiO_2_ is effectively incorporated into the PCL nanofibrous mat to form nanocomposites. The reduced intensities of PCL in PCL-TiO_2_ nanocomposites is because of the presence of physical interaction between PCL and TiO_2_ nanoparticles [38].

### 3.3. Wettability Studies

To evaluate the effect of TiO_2_ nanoparticles on the hydrophilicity of electrospun PCL/TiO_2_ nanocomposites, water contact angles were measured on PCL/TiO_2_ mats and compared to those of cpTi and pure PCL. From Figure 3, it can be noted that a considerable reduction in water contact angle is observed in PCL/TiO_2_ nanocomposite (2, 5, and 7 wt %), indicating improved wetting properties. The water contact angle of the pure PCL sample reached above 140°, demonstrating a hydrophobic surface. Presence of polar surface groups (TiO_2_ nanoparticles) inside the PCL matrix enhanced its hydrophilicity due to a higher interaction between the composite mat and solvent [39]. It is a well-known fact that enhancement in hydrophilicity of a scaffold used in tissue engineering is desirable for initial cell adhesion and cell migration.

### 3.4. Bioactivity Studies

The bioactivity features of PCL/TiO_2_ nanocomposites are well standardized before. Figure 4 presents the SEM images of the cpTi, pure PCL coated and PCL/TiO_2_ nanocomposites with 2, 5 and 7 wt % TiO_2_ content after incubating in SBF for 21 days. The control substrates, i.e., cpTi (Figure 4a), and pure PCL (Figure 4b) exhibit very insignificant mineralization while substantial mineralization takes place in PCL/TiO_2_ nanocomposite samples. It was observed that PCL bio-nanocomposite with higher TiO_2_ concentration i.e., PCL/5TiO_2_ (Figure 4d) and PCL/7TiO_2_ sample (Figure 4e) have the highest nucleation rate of apatite formation after 21 days. It is worth mentioning that even after immersing in SBF for 21 days, the scaffold preserved its microstructure and showed better interconnectivity of the pores.

Development and growth of apatite layer on the substrate is a dynamic process where the material surfaces dissolve and the new bundle of layers precipitates on the surface [40]. When soaked in SBF, the high content of TiO_2_ in PCL/5TiO_2_ and PCL/7TiO_2_ composites leads to an increase of Ti–OH groups on the surface. The unique development of new Ti–OH groups can stimulus the formation of apatite nucleation. Once the apatite nuclei formed, the growth occurs spontaneously by consuming the positive ions of calcium (Ca^2+^) and negative ions of phosphate (PO^3−^) from the SBF fluid to form an amorphous phosphate, which impulsively transforms into hydroxyapatite [Ca_10_(PO_4_)_6_(OH)_2_] [HA] [41]. Based on the EDS analysis for PCL/5TiO_2_ (Figure 4f), the Ca/P molar ratio was estimated to be approximately 1.5 which is in close agreement with the chemical formulation of the biomineral HA [35].

### 3.5. Cytotoxicity-MTT Assay

With the intention of using the PCL/TiO_2_ scaffolds in bone tissue engineering application, the influence of the prepared composites on the growth and viability with hFOB cell lines were investigated for day 1 and day 3 and presented in Figure 5. PCL has widely demonstrated polymer for bone tissue engineering application for its slow degradation kinetics and biocompatibility [42]. In vitro cell culture experiments in the current research manifested the importance of PCL as a function of the TiO_2_ content. In fact, the presence of PCL has enhanced cell proliferation as a function of the TiO_2_ amount. The addition of TiO_2_ nanoparticles at low concentration (2 wt % and 5 wt %) to polymeric PCL scaffolds has favoured the proliferation of hFOB cells. Compared to cpTi and PCL fiber mat, initial attachment and proliferation of PCL/2TiO_2_ and PCL/5TiO_2_ nanocomposites supported the growth of the cells and mediated their proliferation by approximately 20% and 38% respectively. The results demonstrate the substantial and time-dependent growth in cell viability of TiO_2_ at lower concentrations.

However, a noticeable reduction in the cell viabilities is being observed in the PCL/7TiO_2_ sample for both day 1 and day 3. At a higher content of TiO_2_, the TiO_2_ nanoparticles have fetched a significant cytotoxic effect with viabilities of 53 ± 4% and 35 ± 5% as observed on day 1 and day 3 respectively. As we know, the cellular behaviour particle cytotoxicity of TiO_2_ nanoparticles relay on numerous aspects which includes particle shape/size, chemical stability, and mechanical stimulation. Many studies conclusively showed that the effect of TiO_2_ nanoparticles concentrations on cellular behaviour at higher concentrations [25,43]. From the above analysis, it can be implied that the incorporation of TiO_2_ nanoparticles have a positive impact and suggests that PCL/5TiO_2_ can be considered as best suitable biocompatible material that can be employed as a tissue scaffold for the orthopedic application.

### 3.6. Cell Morphology (Cell Adhesion and Proliferation) Studies

**Figure 6:**
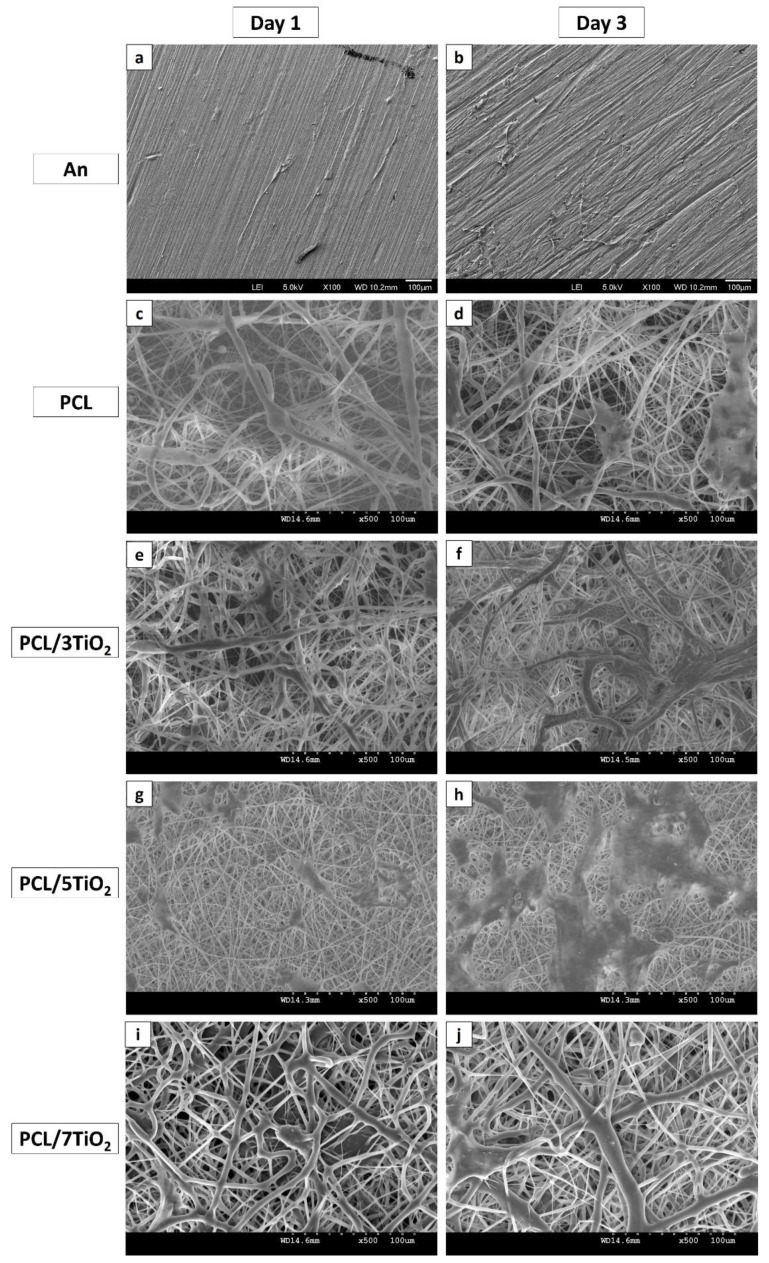
SEM images of hFOB cells seeded on various substrates after day 1, and day 3.

Cell morphology (cell adhesion and proliferation) of An, PCL and PCL/TiO_2_ (2, 5 and 7 wt %) scaffolds were carried out using hFOB, a human osteoblast-like cell lines were presented in Figure 6a–j. Integration of TiO_2_ nanoparticles into PCL, thereby surface coatings on cpTi has significantly increased cell adhesion on day 1 as hFOB cells spread well over the surface and showed the normal morphology of phenotype. Form the SEM analysis, when compared to An (Figure 6a–b) and PCL (Figure 6c–d), it is evident that higher cell proliferation is been observed on the scaffolds containing TiO_2_ nanoparticles. This early interaction of biomolecules and cells with the material is strongly dependent on the PCL/TiO_2_ surface properties, among which hydrophilicity is a key factor. After just 3 days of culture, hFOB cells on PCL/5TiO_2_ nanocomposites showed an excellent homogeneous structure with a clear evidence of cells penetrating into the scaffolds through their pores (Figure 6g–h). The results are well in connection with the wettability studies (Section 3.3). It is evident that the added TiO_2_ nanoparticles increase the surface area and surface hydrophilicity, thus by favoring cell adhesion on day 1 and cell proliferation on day 3.

Interestingly, when compared to other scaffolds, cell behavior to higher TiO_2_ content i.e., with 7 wt % demonstrated little attachment and proliferation (Figure 6i–j). This is probably due to some leachables and an inhomogeneous mixture of TiO_2_ nanoparticles (Figure 1e). Also, it has been stated in many reports that higher TiO_2_ content leads to toxicity into the substrates [25,43,44]. As found in this study, for a given PCL concentration, depending on type of cells used, seeding densities and cell viability test method, it must be definite and established that at given polymeric concentration, the amount of TiO_2_ nanoparticles in scaffolds should not surpass a higher activation level, which should be of the order of 5 wt % in the present case. However, optimized TiO_2_ nanoparticle concentration (5 wt %) will help to engineer the PCL/TiO_2_ based scaffold favoring new tissue formation with an antimicrobial property.

### 3.7. Antibacterial Activity

**Figure 7:**
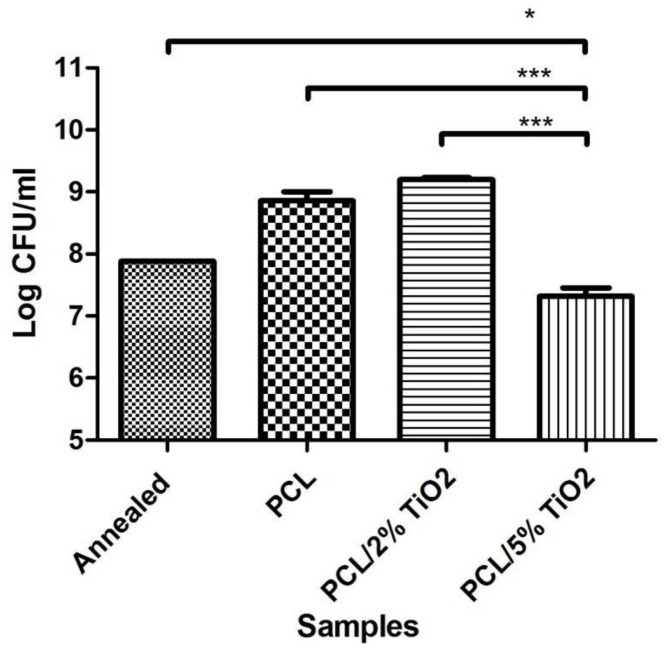
Antibacterial activity on different substrates against *S.aureus*.

Due to the significant toxicity and minimal cell proliferation, the sample with 7% TiO_2_ was eliminated as a candidate from further studies. Amongst the remaining samples, annealed sample with no coating was taken as the control. The electrospun PCL fiber mat shows a relative increase in the bacterial count compared to the control (Figure 7). This is attributed to the fibrous nature of the interface which provides ample opportunities for cellular attachment. This attachment promoting behaviour is also seen in previous studies where the increased surface area contributes to the increase in hFOB proliferation and attachment to electrospun PCL mat and PCL mat containing 2% TiO_2_. This factor proves to be advantageous for bone growth and repair as observed from the study with mammalian cells. However, it may also cause problems due to increased adhesion of bacterial cells. So, it is advisable to use an antimicrobial agent blended with PCL to retard and offset the bacterial growth. TiO_2_ has proven to be such an agent. The antimicrobial properties of TiO_2_ are well known.

**Figure 8:**
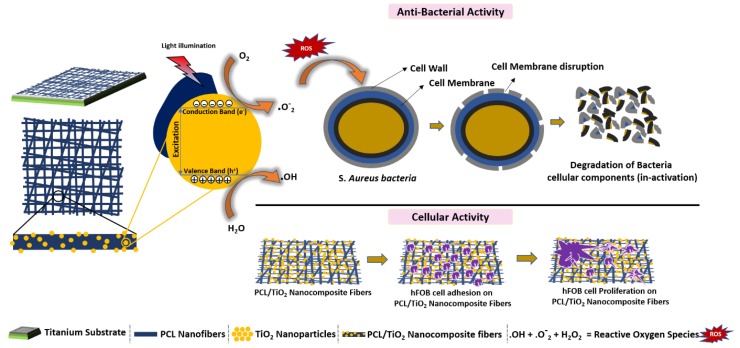
Schematic illustration for the mechanism of degradation bacteria by TiO_2_ nanoparticles under UV radiation and cellular activity of PCL/TiO_2_ nanocomposites.

As presented in Figure 8, several fundamental mechanisms for cell killing and bacterial growth inhibition by the TiO_2_ photocatalytic processes were presented in the literature [45,46]. As a result the blend of PCL and 2% TiO_2_ was initially tested against *S.aureus*. However, there is no significant difference between the PCL fiber mat and the PCL fibers containing 2% TiO_2_. This implies that this concentration of TiO_2_ is not enough to bring about a photocatalytic effect that reduces the bacterial count. On increasing the TiO_2_ concentration to 5%, significant reduction is seen in the bacterial colony count compared to plain annealed sample (control). Thus 5% TiO_2_ also seems to be the minimal concentration necessary to be incorporated with PCL to achieve antibacterial activity. It also appears to be the optimal concentration of TiO_2_ necessary for to give an antibacterial action without significant toxicity. This nanocomposite thus proves to be an efficient approach to achieving bioactivity and improving integration of the titanium implants.

## 4. Conclusions

We report the fabrication of functional PCL-TiO_2_ coatings by means of the electrospinning technique and coated on cpTi substrates for bone tissue engineering. A range of electrospun nanocomposite scaffolds were fabricated with a novel and simple material consisting of biodegradable polymer PCL (8 wt %) and TiO_2_ (2, 5 and 7 wt %). When subjected to SBF, the enrichment of a PCL scaffolds was observed when TiO_2_ nanoparticles were incorporated into it. The TiO_2_ nanoparticles serves as a basis of inorganic phosphate to enhance bone cell mineralization, demonstrating the bioactive features of the hybrid scaffolds. The results obtained by hybrid PCL/TiO_2_ coatings have a cell viability more than the uncoated one and pure PCL suggesting the effect of nanoparticles. On the contrary, uncoated cpTi and PCL/7TiO_2_ appears to be less biocompatible mainly due to minimum contact between the cells-substrate and toxic effect of higher TiO_2_ content respectively. The synthesized nanocomposite PCL/TiO_2_ fibers possess the capacity to replicate surface chemical properties of bone and also acts as an antibacterial surface with greater efficacy and performance under strong UV radiation. In particular, PCL/5TiO_2_ coatings mimic the excellent biological response with respectable antibacterial activity. Because of the effective antibacterial property, no additional covering of the wound for infection prevention is required. The obtained coatings thus can be successfully used to change the surface of cpTi implants to improve their biological properties and antibacterial performance.
